# MRI susceptibility map weighted imaging (SMWI) as a neurodegeneration biomarker in the prodromal to overt alpha-synucleinopathy continuum

**DOI:** 10.1177/1877718X251387027

**Published:** 2025-12-03

**Authors:** Laura Falcitano, Francesco Calizzano, Pietro Mattioli, Oliver C Kiersnowski, Laura Avanzino, Nicola Giovanni Girtler, Andrea Diociasi, Mattia Losa, Federico Massa, Silvia Morbelli, Beatrice Orso, Elisa Pelosin, Gaia Bonassi, Stefano Raffa, Matteo Pardini, Mauro Costagli, Luca Roccatagliata, Dario Arnaldi

**Affiliations:** 1 9246 IRCCS Ospedale Policlinico San Martino, Genova, Italy; 2 Department of Neuroscience, Rehabilitation, Ophthalmology, Genetics, Maternal and Child Health, 159128 University of Genova, Genova, Italy; 3 198209 Department of Experimental Medicine, University of Genova, Genova, Italy; 4 Department of Internal Medicine and Medical Specialities (D.I.M.I.), 215995 University of Genoa, Genoa, Italy; 5 Division of Nuclear Medicine, Department of Medical Sciences, 18691 University of Turin, Turin, Italy; 6 165481 Department of Health Sciences, University of Genova, Genova, Italy

**Keywords:** alpha-synuclein, biomarkers, REM sleep behavior disorder, MRI swallow tail sign, dat-SPWECT neurodegeneration, susceptibility weighed imaging

## Abstract

**Background and objective:**

Nigrostriatal dopaminergic degeneration is commonly assessed using dopamine transporter (DaT) SPECT. Iron sensitive MRI is a promising technique to assess substantia nigra, yet few studies explored its application in the prodromal stage of alpha-synucleinopathies. Here, we used susceptibility map weighted imaging (SMWI) to assess the swallow tail sign, a radiological marker for substantia nigra integrity, to detect neurodegeneration across the alpha-synucleinopathy continuum.

**Methods:**

3T-MRI was performed on 115 subjects: 27 overt alpha-synucleinopathies, 34 prodromal alpha-synucleinopathies, 28 Alzheimer's disease and 26 healthy controls. SMWI was obtained with 3D multi-echo gradient-echo imaging. The presence/absence of the swallow tail sign was visually evaluated on SMWI by two neuroradiologists, blinded to the diagnosis. Swallow tail sign's visual assessment was compared across groups to investigate its sensitivity and specificity in identifying alpha-synucleinopathies. Additionally, we compared the SMWI visual analysis sign with both substantia nigra quantitative susceptibility mapping (QSM) and DaT-SPECT.

**Results:**

The two radiologists’ inter-rater agreement was substantial (kappa = 0.8). Visual analysis showed good sensitivity (0.85) and specificity (0.82) in identifying patients with alpha-synucleinopathies. When the subjects were grouped based on DaT-SPECT results, sensitivity increased (0.92), while specificity decreased (0.74). Visual scoring was associated with quantitative substantia nigra MRI assessment obtained with QSM (p < 0.001). Lastly, subjects with the swallow tail sign rated as absent showed significantly lower (p = 0.019) uptake at DaT-SPECT (−1.857 ± 1.343) compared to those with the swallow tail sign rated as present (−0.385 ± 1.850).

**Conclusions:**

Visual analysis of SMWI swallow tail sign represents a new and reliable approach for evaluating substantia nigra neurodegeneration across the alpha-synucleinopathy continuum.

## Introduction

Alpha-synucleinopathies are disabling neurodegenerative diseases, with the most common clinical phenotypes being Parkinson's disease (PD) and dementia with Lewy Bodies (DLB).^1,2^ Neurodegeneration in alpha-synucleinopathies is typically assessed by evaluating nigrostriatal pathway dysfunction^3^ using single photon emission computed tomography (SPECT) with [^123^I]-ioflupane (DaT-SPECT) which serves as the gold standard for in vivo evaluation of the nigrostriatal pathway.^4^ Less invasive neuroimaging techniques to evaluate nigrostriatal pathway are currently under validation in research settings. For example, iron sensitive MRI can provide with promising markers of neurodegeneration to assess the substantia nigra (SN)^2^; however, this approach is still considered investigational by the recent biological research frameworks,^1,2^ with few studies exploring its use in prodromal alpha-synucleinopathies.^5–8^ A visually assessable radiological marker of SN pathology is the loss of the physiological dorsal nigral hyperintensity (DNH), which is caused by iron accumulation. DNH is typically evaluated using magnetic resonance susceptibility-weighted imaging (SWI) a technique combining the magnitude and phase images obtained with a gradient echo (GRE) acquisition. In particular, the SWI reconstruction uses the signal phase, reflecting local field inhomogeneities originating from biological tissues’ magnetic susceptibility, to enhance the T2*-weighted contrast embedded in the conventional magnitude images. This way, the hypo-intensity of paramagnetic tissues, such as those with increased iron concentration, is emphasized. The normal appearance of the DNH in SWI at the expected anatomical location of nigrosome 1 (N1) at level of the midbrain level is known as the swallow tail sign.^9–13^ The assessment of the swallow tail sign using SWI in MRI scanners operating at 3T showed excellent sensitivity (94.6%) and specificity (94.4%) in identifying patients with overt PD^3^ but only fair sensitivity (63%) and specificity (79%) in identifying DLB.^14^ Few studies investigated iron sensitive MRI in the prodromal phases, such as in idiopathic/isolated REM sleep behavior disorder (iRBD), showing abnormal N1 imaging in about 60% of iRBD cases.^7^ The identification of reliable biomarkers able to assess substantia nigra neurodegeneration since prodromal alpha-synucleinopathy stage is of paramount importance in the light of the new framework for a biological definition of PD, as well as upcoming clinical trials testing disease modifying drugs.

One known limitation of SWI is that it directly relies on local field inhomogeneities to emphasize magnetic susceptibility effects: such field inhomogeneities spatially extends beyond the tissues that generate them, therefore the image feature emphasized by SWI do not precisely co-localize with the underlying anatomy. An improved implementation of SWI, namely susceptibility mapping weighted imaging (SMWI), relies on the same acquisition as SWI, but employs a refined reconstruction pipeline including an image processing step to remove the non-local phase perturbations by a deconvolution operation.^15^ Therefore, SMWI results in improved susceptibility contrast and reduced “blooming” artifact^15^ and has been proved to provide enhanced N1 anatomical depiction and visibility at 3T when compared to conventional SWI. A recent multi-centric study involving patients with degenerative and non-degenerative forms of parkinsonisms reported no significant difference between SMWI and DaTPET diagnostic performance.^16^ SMWI has never been tested in prodromal stages of alpha-synucleinopathies and we hypothesized that SMWI would enable neuroradiologists detecting an altered appearance of the substantia nigra not only in the overt forms of alpha-synucleinopathy, but also the prodromal stages.

The aim of the present project was to confirm this hypothesis by visually evaluating the SMWI swallow tail sign representations of patients along the prodromal to overt alpha-synucleinopathy continuum. For comparison, we included a group of healthy subjects (HC) and patients with Alzheimer's disease (AD). Moreover, we assessed the relationship between the SMWI visual evaluation of the swallow tail sign and quantitative susceptibility mapping (QSM) semi-quantification of iron content in the substantia nigra to further validate this imaging marker. Finally, we aimed to compare the SMWI assessment with DaT-SPECT results.

## Materials and methods

### Patients

This project is a single center prospective case-control study conducted between November 2020 and February 2024. The main inclusion criteria were the diagnosis of PD,^17^ DLB,^4^ prodromal DLB (pDLB)^18^ or iRBD,^19^ determined in accordance with current criteria by a movement specialist for PD, a cognitive specialist for DLB and pDLB, and a sleep specialist for pDLB and iRBD. The diagnoses of PD and DLB were further confirmed through clinical follow-up of at least 12 months. To be consistent with the new concept of the alpha-synucleinopathy continuum,^1,2^ PD and DLB patients were classified as having overt alpha-synucleinopathy, while iRBD and pDLB patients were considered prodromal stages of alpha-synucleinopathy. All subjects underwent 3 T brain MRI; additionally, patients with overt DLB and all prodromal patients also underwent brain DaT-SPECT.

As healthy controls, we enrolled a group of subjects with no history of neurological, cognitive or psychiatric disorders (including but not limited to depression and anxiety). Moreover, we included a group of pathological controls diagnosed with AD at the MCI or dementia stage, according to current criteria.^20^ All AD patients met the criteria for probable AD,^20^ with at least intermediate likelihood based on clinical and neuronal injury imaging data (MRI and/or [18F]FDG PET]. Moreover, imaging and clinical data were carefully assessed to exclude the potential misclassification of DLB patients in the AD cohort. Furthermore, 71.4% of them were considered at high likelihood of AD^20,21^ in presence of positive amyloid biomarker according to PET with specific tracers or cerebral spinal fluid assessment of amyloid isoforms (Aβ42/Aβ40 ratio), also in accordance with the proposed AT(N) research framework.^22^

Exclusion criteria were claustrophobia, or any contraindications to MRI. MRI scans with motion artifact that precluded visual analysis were not included.

### Ethics statement

The study protocol was approved by the local Ethics Committee, and an Informed consent form was signed by all participants, in compliance with the Helsinki Declaration of 1975.

### Magnetic resonance imaging

#### MRI acquisition

All subjects were scanned with a Siemens Prisma 3 T MR system equipped with a 64-channel head coil. The protocol included whole-brain acquisitions of magnitude and phase data using a multi-echo 3D gradient echo (GRE) sequence with eight echoes at TE1/ΔTE = 5.6/5.6 ms; TR = 51 ms; flip angle 18°; 1 × 1 × 1 mm3 resolution; 224 × 224 × 144 matrix size; GRAPPA = 2; partial Fourier 6/8 in both Phase-encoding (PE) directions; Bandwidth (BW) = 340 Hz/px; adaptive coil combine with pre-scan normalize on; acquisition time = 8 min 45 s.^23,24^

Additional structural brain images were acquired in the sagittal plane using a T1-weighted MPRAGE with TR = 2300 ms; TE = 2.96 ms; TI = 900; flip angle 9°; 1 × 1 × 1 mm^3^ resolution; 256 × 256 Matrix size; GRAPPA = 2; BW = 240 Hz/px; acquisition time = 5 min 30 s.

#### MRI data processing

For each patient, R2* map, QSM and SMWI were reconstructed, as detailed below. All image reconstructions were carried out using Matlab 2019b (MathWorks, USA).

R2* maps were calculated via the Auto-Regression of Linear Operations (ARLO) method^25^ from the MEDI Toolbox (http://pre.weill.cornell.edu/mri/pages/qsm.html).

QSM maps were reconstructed according to the RIN-network's protocol previously described in detail^24^ using a processing pipeline based on STI Suite (https://people.eecs.berkeley.edu/∼chunlei.liu/software.html from UC Berkeley, Berkeley, CA, USA). In short, a brain mask was calculated from the T2*-weighted magnitude image averaged across all echoes using the Brain Extraction Tool (BET) from FSL.^26^ For each echo, raw phase images were unwrapped using Laplacian phase unwrapping^27^ followed by background field removal using the Variable-kernel Sophisticated Harmonic Artifact Reduction for Phase data (V-SHARP) approach.^28^ QSM maps were then calculated using the iLSQR method.^24,29^

SMWI was obtained by merging each subject's GRE magnitude image averaged across all echoes and QSM according to a previously documented pipeline^15^ with parameters X_th_ = 1 part per million and m = 4.

The whole SN was segmented via MRICloud with the “multi-atlas tool for automated segmentation of brain grey matter nuclei and quantification of their magnetic susceptibility^30,31^ based on the joint contrast information from the co-registered T1-weighted image and susceptibility map. The SN segmentations were then eroded via a convolution with a 1 mm Gaussian kernel to avoid partial volume effects. This tool was reported to perform better than that of manual delineation as described in detail by Li X et al.^30^ The schematic representation of the MRI data processing pipeline is shown in Supplementary Figure 1 and an explanatory example of the segmentation process is shown in Supplementary Figure 2.

#### MRI image scoring

**Figure 1.**
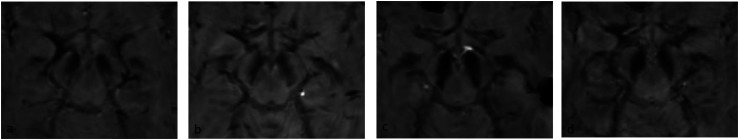
Examples of swallow tail sign. From left to right, present (a), probably present (b), probably absent (c), absent (d). Of notice, the presence of a swallow tail sign is a normal finding in healthy subjects, whereas the absence of the sign is considered as an abnormal finding consistent with substantia nigra degeneration in alpha-synucleinopathy.^14,32^.

SMWI reconstructions were evaluated to assess the SN at the level of the posterior third part below the red nucleus using a validated scale of preservation of the swallow tail sign (dorso nigral hyperintensity, DNH) corresponding to the anatomical location of N1 based on trilaminar aspect of SN. In detail, the sign received a score ranging from 1 to 4 (1 = present, 2 = probably present, 3 = probably absent, 4 = absent as shown in Figure 1).^14^ Visual scores were subsequently dichotomized as present (1 and 2) and absent (3 and 4) and this scoring system was applied independently to right and left nigrosome by two expert neuroradiologists (LF, LR), blinded to the diagnosis of the subject. In the case of different scoring, the case was evaluated again, a consensus reached, and the worst side defined in each patient. To note, a present swallow tail sign is thought to reflect a normal SN, while an absent sign is indicative of an abnormal SN.

### DaT-SPECT

DaT-SPECT was acquired according to EANM guidelines^33^ in the subgroup of patients diagnosed with prodromal alpha-synucleinopathies and in overt DLB patients, within 12 months since MRI. Data were acquired by means of a 2-headed Millennium VG camera (G.E. Healthcare), as previously described.^34^ The reconstructed DaT-SPECT images were processed using the DaTQUANT^TM^ v 2.0 software to obtain specific binding ratios (SBR) for putamen and caudates nuclei as previously reported.^35^

Putamen's DaT-SPECT z-scores were calculated, in agreement with the literature.^36^ The z-scores of the most affected putamen (MAP) were further categorized as below or above the cut-off of −1. We choose the cut-off of −1 at MAP because it was found to be the most accurate in identifying iRBD patients at high risk of short-term phenoconversion.^36^ Accordingly, for the statistical analysis prodromal patients were considered as positive (prodromal positive, PP) or negative (prodromal negative, PN) depending on the MAP Z score (i.e., below/above −1 respectively). In this case, a positive DaT-SPECT reflects an impaired nigro-striatal dopaminergic function, while a negative DaT-SPECT reflects a normal function.

### Statistical analysis

Firstly, we performed a descriptive analysis. For normally distributed data, we used analysis of variance (ANOVA), t-tests for continuous variables, and chi-squared statistics for categorical variables. For non-normally distributed data, Kruskal-Wallis and Mann-Whitney test were performed.

We explored the agreement between the two raters by performing a Cohen's Kappa agreement test. Subsequently, we computed the rates of present/absent swallow tail sign at SMWI visual inspection in all enrolled subjects, as well as the sensitivity and specificity of the swallow tail sign in detecting alpha-synucleinopathies. We used the clinical diagnosis as ‘ground truth’, and considered the positive and negative results of the nigrosome assessment as follows:

- A true positive was considered when the swallow tail sign was rated as absent (abnormal) and the patient had either prodromal or overt (either DLB or PD) alpha-synucleinopathy,
- A true negative was defined as when the swallow tail sign was rated as present (normal) and the patient was diagnosed with either AD or was a healthy control,
- A false positive occurred when the swallow tail sign was rated as absent (abnormal), but the subject was diagnosed with either AD or classified as a healthy control,
- A false negative was identified when the swallow tail sign was rated as present (normal), but the patient had either overt or prodromal alpha-synucleinopathy.

However, prodromal subjects may exhibit either a positive or negative DaT-SPECT, which has relevant prognostic implications. Specifically, patients with iRBD who have a positive DaT-SPECT have a high risk of short-term phenoconversion.^35^ To effectively group subjects with and without nigrostriatal impairment, we conducted a secondary analysis, where prodromal patients with a negative DaT-SPECT (i.e., MAP z score above −1) were assigned to the control group, which also included HC and AD patients. Conversely, prodromal patients with an altered DaT-SPECT (i.e., MAP z score below −1) were assigned in the study group, alongside overt PD and DLB patients. This secondary analysis allowed us to compute the sensitivity and specificity of the SMWI swallow tail sign assessment in identifying alpha-synucleinopathy patients with nigrostriatal dopaminergic dysfunction, compared to subjects with an intact nigrostriatal dopaminergic pathway, namely AD patients, healthy controls and prodromal patients with a negative DaT-SPECT at the time of the evaluation.

To explore whether the visual analysis of the swallow tail sign corresponded with quantifiable changes in the SN, we then compared mean magnetic susceptibility (from QSM) and R2* across the groups defined by visual scores of the swallow tail sign. To achieve this, we applied two general linear models (GLMs) to assess susceptibility/R2* in relation to visual scores, while including age as a confounding factor, with a significance level of 0.05.

Finally, we compared the DaT-SPECT results with the visual scores of the swallow tail sign. For this analysis, DaT-SPECT z-scores were categorized dichotomously (i.e., below or above −1 z-score).

Statistical analysis was performed with R studio implemented in Bluesky statistics (https://www.blueskystatistics.com), and Python (https://www.python.org).

The data used in the present study can be requested upon reasonable request.

## Results

Out of 130 subjects that underwent MRI, 15 were excluded by the study because of motion artifacts whereas 115 subjects were enrolled (mean age = 70.78 ± 8.50, 48.69% females): (i) 34 patients in the prodromal stage of alpha-synucleinopathy (mean age = 72.35 ± 7.25, 32.35% females; 21 iRBD, 13 pDLB), of which 12 with a MAP z-score above −1 (prodromal negative group, PN, mean age = 71.08 ± 7.87, 33.33% females) and 22 with a MAP z-score below −1 (prodromal positive group, PP, mean age = 73.05 ± 6.98, 31.81% females), (ii) 27 patients diagnosed with overt alpha-synucleinopathy (mean age = 69.07 ± 7.91, 44.44% females, 22 PD, 5 DLB), (iii) 26 healthy subjects (mean age = 69.89 ± 10,90, 73.07% females), and (iv) 28 patients diagnosed with AD (mean age = 71,35 ± 7.98, 50% females). Demographic and clinical data of the patients are reported in Table 1.

**Table 1.** Demographic and clinical data of the whole group.

	Healthy Controls n = 26	Prodromal aSyn n = 34		Overt aSyn n = 27		AD N = 26
Diagnosis	n/a	RBD n = 21	pDLB n = 13	PD n = 22	DLB n = 5	n/a
Age (mean; SD)	69.88; 11.10	72.35; 7.34		69.07; 7.61		71.36; 7.97
Sex (Females)	19 (73.08%)	11 (32.35%)		12 (44.44%)		14 (53.85%)
Mini Mental State Examination (mean; SD)	n/a	27.32; 3.02		26.00; 4.84		23.72; 3.50
MDS-UPDRS III (mean; SD)	n/a	6.20; 6.76		19.85; 10.84		n/a
Levodopa equivalent daily dose (LEDD) (mean; SD)	n/a	n/a		PD 620.30 mg; 161.49 mg	DLB n/a	n/a

RBD: REM Behavioral Disorder; pDLB: prodromal dementia with Lewy bodies; PD: Parkinson's disease; DLB: Dementia with Lewy bodies; SD: standard deviation.

### Inter-rater agreement

The two raters demonstrated substantial agreement (K = 0.80, Standard error (SE) = 0.03, Confidence interval (CI) =0.75–0.85) in scoring the swallow tail sign within one of the four classes provided. More in detail, the rate of disagreement was 22.94% in the whole group of nigrosomes (left + right, 53 out of 230), 16.67% in the control group (18 out of 108, 5.77% in the healthy control group (3 out of 52), 26.79% in the AD group (15 out of 56)), 26.47% in the prodromal group (18 out of 68) and 31.48% (17 out of 54) in the overt group. When considering the dichotomized classes (present/absent) the two raters demonstrated substantial agreement (K = 0.82, Standard error (SE) = 0.04, Confidence interval (CI) = 0.75–0.90) and the rate of disagreement decreased as follows: 6.09% (14 out of 230) in the whole group, 9.26% (10 out of 108, 5.77% in the healthy control group (2 out of 52), 14.29% in the AD group (8 out of 56)) in the control group, 5.88% (4 out of 68) in the prodromal group, 0% (0 out of 54) in the overt group. Further analyses were performed based on the consensus of the two neuroradiologists regarding the most affected side.

### Presence/absence of the swallow tail sign across groups

**Figure 2.**
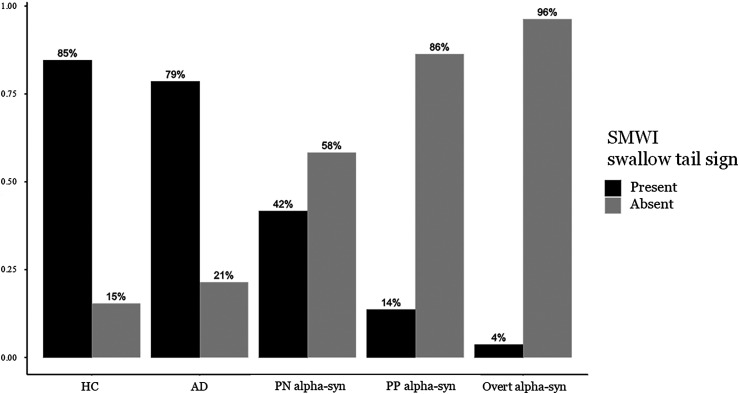
Relative frequencies of the presence and absence of the swallow tail sign in SMWI in the most affected side were analyzed in: healthy controls, patients affected by AD, patients with prodromal alpha-synucleinopathy and overt alpha-synucleinopathy. The prodromal group was further divided based on their most affected putamen z-scores, with one subgroup above the cut-off (prodromal negative PN alpha-syn), and the other below the cut-off (prodromal positive alpha-syn). Chi-squared test results for comparison: HC vs. AD =0.568, HC vs. PN alpha-syn <0.007, HC vs. PP alpha-syn <0.001, HC vs. Overt alpha-syn <0.001, AD vs. PN alpha-syn =0.022, AD vs. PP alpha-syn <0.001, AD vs. Overt alpha-syn <0.001, PN alpha-syn vs. PP alpha-syn =0.066, PN alpha-syn vs. Overt alpha-syn <0.002, PP alpha-syn vs. Overt alpha-syn =0.207. Legend: AD: Alzheimer disease; PN alpha-syn: Prodromal Negative alpha-syn; PP alpha syn: Prodromal Positive alpha-syn.

Rates of present/absent swallow tail sign were different among groups (p < 0.001, Figure 2). Alpha-synucleinopathies, whether in their prodromal or overt stage, had a higher proportion of absent swallow tail sign and a lower proportion of present swallow tail sign compared to both HC and AD patients.

Notably, prodromal patients with negative DaT-SPECT appear to fall between control subjects and those with overt alpha-synucleinopathy, exhibiting a relatively balanced percentage of swallow tail sign presence/absence scores (42% versus 58%, respectively). In contrast, prodromal patients with positive DaT-SPECT had swallow tail sign ratings comparable to those of overt alpha-synucleinopathy patients, as shown in Figure 2.

### Sensitivity and specificity assessment

The SMWI visual analysis of the swallow tail sign showed a good sensitivity (0.85) and specificity (0.82) in correctly identifying patients with alpha-synucleinopathy, along with a good accuracy, and positive and negative predictive values (0.83, 0.84, and 0.83, respectively).

In a secondary analysis, where prodromal patients were categorized according to the DaT-SPECT results, the SMWI visual analysis of the swallow tail sign showed an increase in sensitivity and negative predictive value (both 0.92), a decrease in specificity and positive predictive value (0.74, 0.73, respectively), while accuracy remained nearly unchanged at 0.82.

### Quantitative MRI compared to visual scores

**Figure 3.**
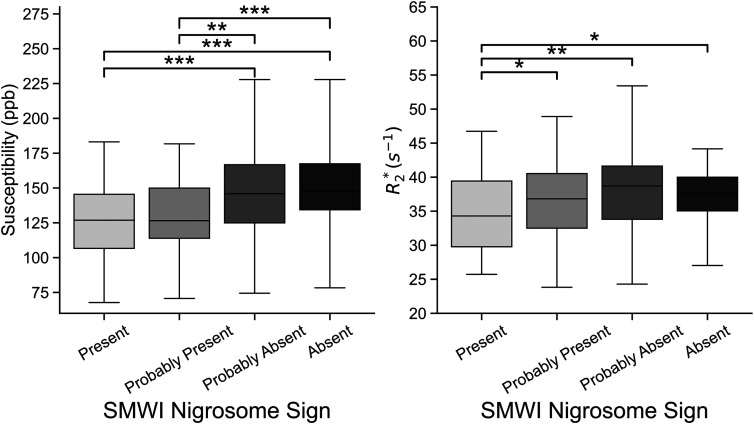
Boxplots of mean susceptibility (left) and R2* (right) in the substantia nigra (SN) based on categorical SMWI visual ratings of the swallow tail sign. FDR-corrected post-hoc t-tests revealed no differences in susceptibility between groups 1 and 2 (presence of swallow tail sign), and between scores of 3 and 4 (absence of swallow tail sign). However, there was a significant increase in R2* between score 1 and scores 2, 3, or 4. Scores of 3 and 4 exhibited significantly higher susceptibility than both scores of 1 and 2. * indicates p < 0.05, ** indicates p < 0.01 and *** indicates p < 0.001; ppb = parts per billion.

GLMs of SN susceptibility and R2* as functions of SMWI visual rating and age were significant, indicating that both predictors significantly impacted susceptibility and R2* (F = 10.499, p < 0.001; F = 6.919, p < 0.001, respectively). Even if a certain overlap in terms of confidence interval was observed, post-hoc t-tests between visual ratings revealed significant differences in mean SN susceptibility and R2* based on visual scores, as summarized in Figure 3. Of notice 4 scans (2 HC, 1 AD, 1 RBD) were affected by mild movement artifacts or presented acquisition incompatibility with the analysis, hence were removed from the quantitative analysis.

### Comparison between DaT-SPECT and swallow tail sign SMWI visual evaluation in prodromal patients

Thirty-eight patients underwent DaT-SPECT imaging. Among these, 4 patients had overt DLB (mean age = 76.40 ± 7.44, 40% females), while 34 patients had prodromal alpha-synucleinopathy (mean age = 72.35 ± 7.25, 32.35% females). In this subgroup, the rates of positive and negative findings for the MAP and swallow tail sign were associated, although this association did not reach statistical significance (p = 0.066). However, when considering DaT-SPECT values as a continuous variable, subjects with the swallow tail sign rated as absent showed significantly lower (p = 0.019) DaT-SPECT values (−1.857 ± 1.343) compared to those with the swallow tail sign rated as present (−0.385 ± 1.850).

## Discussion

Recently proposed research frameworks for the diagnosis of neuronal alpha synuclein disease are grounded on the identification of the biological substrate of the disease and of the nigro-striatal dopaminergic disfunction and envision the use of biomarkers to stage neuronal alpha-synuclein disease and detect neurodegeneration.^1,2^

Currently, DaT-SPECT is widely used for nigro-striatal pathway assessment.^33^ Specifically, the binding ratio in the MAP, adjusted for age and sex, is used to categorize individuals as having or not having a dopamine transporter deficit.^2^ Since the new alpha-synucleinopathy research framework^1,2^ allows for the incorporation of emerging measures of dopaminergic dysfunction, in addition to or instead of dopamine transporter loss, the validation of new biomarkers reflecting neurodegeneration specific to neuronal α-synuclein disease remains a current challenge.

In this context, susceptibility weighted imaging has emerged as a promising tool for identifying biomarkers of nigrostriatal neurodegeneration.^3,5^ Indeed, several studies have demonstrated increased quantitative magnetic susceptibility in the SN as estimated by QSM in patients with PD,^37,38^ which has been shown to be a consequence of iron accumulation in this region.^39^

In this study, we assessed the visual evaluation of the nigrosome using susceptibility map weighted imaging (SMWI) to detect SN impairment in a large and heterogeneous cohort of patients along the continuum from prodromal to overt alpha-synucleinopathy.

SMWI employs a susceptibility weighting mask derived from QSM that enhances the visibility of the swallow tail sign and reduces the “blooming” artifact,^15^ whose high diagnostic performance in determining nigrostriatal degeneration was demonstrated on different MRI scanners in a recent multi-centric study.^16^ In this study, SMWI was reconstructed from the 3D GRE 8-echo acquisition included in the harmonized protocol of the” Italian Neuroscience and Rehabilitation Network” as described in Lancione M et al.^24^ with an acquisition time of more than eight minutes which could be difficult to implement in the clinical setting. It is worth pointing out that the most recent international recommendations^40^ suggest the use of fewer echoes with shorter maximum echo time, which enables shorter acquisition times. Superb SMWI quality can be achieved also with even more efficient acquisition schemes, such as echo-planar imaging, as recently demonstrated in the context of a different neurological application.^41^

Using a visual assessment score on SMWI, we demonstrated that both patient groups in the prodromal and overt stages of the disease exhibit a high rate of absence of the swallow tail sign (96% in overt alpha-synucleinopathies).

When the clinical diagnosis was used as the ‘ground truth’, the sensitivity and specificity of the SMWI swallow tail sign visual assessment in identifying alpha-synucleinopathy patients were good (above 0.8).

However, when grouping the subjects based on nigrostriatal dopaminergic deficit, both the sensitivity and the negative predictive value improved to excellent (above 0.9), while the specificity and the positive predictive value became acceptable (above 0.7). Additionally, the visual assessment of the SMWI swallow tail sign was associated with quantitative changes in magnetic susceptibility in the SN as measured by QSM, indicating a substantial overlap between visual assessment and semi-quantified approach.

We found that the visual assessment of the SMWI swallow tail sign is highly reproducible, showing a substantial inter-rater agreement (K = 0.8). Such results suggest that in the study of neuronal alpha synuclein diseases, SMWI might be potentially useful to detect SN neurodegeneration in fulfillment of recently proposed biological definition of alpha-synucleinopathies,

The prodromal stages of the disease have been less thoroughly investigated using iron-based MRI. Given that SN degeneration has been proposed as a predictor of phenoconversion to overt alpha-synucleinopathy,^32^ its early recognition is of paramount importance.

Even if some research has been performed in the imaging of iron accumulation and in particular on SWI in PD^10–13^ the literature on the prodromal stages of alpha-synucleinopathies is overall inconsistent. It showed a loss of DNH in 25%^6^ to up to 61% of iRBD cases,^5,7^ depending on the magnetic field strength and the MRI scan sequences. Our results outperform those of previous studies, showing that in 77% of prodromal patients, the swallow tail sign was scored as absent. Notably, in our sample, approximately 60% of prodromal subjects with a negative DaT-SPECT had the swallow tail sign scored as absent, while in up to 86% of prodromal patients with a positive DaT-SPECT also had the swallow tail sign scored as absent. This finding further strengthened the hypothesis that SMWI may be a reliable biomarker for SN neurodegeneration since prodromal alpha-synucleinopathy stage, with high negative predictive value.

The SMWI visual assessment of the swallow tail sign successfully distinguished the prodromal stages of the disease from healthy subjects, even in the absence of a positive DaT-SPECT. This supports the idea that the loss of DNH reflects the accumulation of iron that occurs in the very early stages of the disease, suggesting that the morphological abnormalities detectable by SMWI at the SN level may manifest before functional presynaptic dopaminergic impairment can be detected by the DaT-SPECT.^42^ Conversely, no significant difference was found between overt alpha-synucleinopathies and prodromal patients with positive DaT-SPECT, suggesting that iron deposition in the SN begins in the prodromal phase of the disease and reaches a floor effect in the “late” prodromal/early overt stages, hindering the identification of significant differences. Indeed, while the SMWI swallow tail sign visual assessment and DaT-SPECT were associated, there was not a complete overlap, suggesting that these two metrics reflect different, though connected, aspects of the substantia-nigra dopaminergic system. In fact, recent evidence suggests that assessing the terminal part of the nigrostriatal pathway does not necessarily reflect the condition of the neuronal cell bodies and/or axons^43–45^ reported the presence of scans without evidence of dopaminergic deficit (SWEDDs) in a series of eight elderly patients with clinical Parkinson's disease. They speculated that this finding might be related to lower striatal compensatory mechanisms in late-onset PD.^45^ Given the mean age of our prodromal group (72.35 ± 7.25 years), it is possible that a similar mechanism may be at play in part of our cohort as well. This possibility strengthens the hypothesis that visual assessment of the SMWI swallow tail sign could serve as a valuable biomarker for identifying neurodegeneration, even in the prodromal stages of alpha-synucleinopathies, by directly evaluating neuronal body degeneration. Overall, these findings suggest that the SMWI swallow tail sign visual assessment may serve as a valuable biomarker for identifying neurodegeneration, applicable from the prodromal stages of alpha-synucleinopathy, and possibly providing, in a small proportion of patients, non-redundant information compared to DaT-SPECT. Interestingly, 85% of healthy controls showed a present SMWI swallow tail sign (normal), while 79% of AD patients were rated as normal. Although this difference did not reach statistical significance, it may reflect the presence of alpha-synuclein-related co-pathology^46^ in a subset of AD patients and might have affected the performances of the neuroimaging techniques. In fact, in a recent study on 240 biologically determined AD patients, a positive αSyn Seed Amplification Assay, suggestive for the presence of co-pathology, was disclosed in 30% of the patients,^46^ in agreement with pathological studies.^47^

We did not assess our patients with biological markers of alpha synuclein (eg SAA on different biological tissues) and this can be a limitation, considering the known possibility of the presence of co-pathology as previously described.^48^

It is worth pointing out that the AD patients enrolled in the present study did not have any clear sign of parkinsonism, but patients did not undergo systematic motor symptom evaluation. Thus, mild motor symptoms may have been present but unreported. Moreover, it would be particularly interesting to follow the 15% of healthy subjects with an absent SMWI swallow tail sign (abnormal) over time to evaluate whether they might develop signs related to alpha-synucleinopathy. Notably, the healthy subjects did not report dream-enacting behaviors; however, they did not undergo polysomnography, so we cannot exclude the presence of subclinical RBD, as a very early, prodromal, sign of alpha-synucleinopathy. Other, non-specific-motor symptoms commonly seen in the prodromal stages of synucleinopathies, such as hyposmia and constipation, were not formally assessed.

To quantitatively assess the swallow tail sign visual, we employed QSM. This method has not been widely evaluated in iRBD patients.^49^ However, voxel-wise analysis of QSM revealed increased susceptibility within the SN in both PD and iRBD compared with HC.^50^ There is a growing interest in QSM as a progression biomarker in prodromal alpha-synucleinopathies.^32^ Indeed, both paramagnetic and diamagnetic components of susceptibility have recently been shown to carry important information reflecting dopaminergic nigrostriatal degeneration.^8^ Another study focusing on prodromal stages employed a method of signal quantification that did not use QSM but yielded similar results.^6^ In this study, we found a significant difference in susceptibility and R2* in the SN across the four classes of visual rating. These findings provide a physical basis for the visual analysis of SMWI nigrosome representation, possibly offering neuroradiologists, after further amelioration and validation of the methodology, an easier-to-use yet accurate tool for assessing nigrosome integrity.

Finally, while the swallow tail sign has been widely investigated on iron-sensitive imaging, other techniques can be considered as potential biomarkers of the disease; for instance, diffusion-weighted MRI and specifically free-water imaging^51^ and neuromelanin sensitive MRI. In particular, it has been found that the posterior region of the substantia nigra, below the level of the red nucleus, is the most sensitive in terms of classifying PD and predicting disease progression^52,53^ and the current findings support the interpretation of posterior SN free water values provides a marker for the relative state of the substantia nigra.^54^ On the other hand, neuromelanin-sensitive-MRI gauges intracellular neuromelanin accumulation leveraging on T1 reduction effect of neuromelanin and has been proposed as a marker of neuronal degeneration in the SN.^55^

Our results align with the potential association between changes in the swallow tail sign appearance and free water changes in the posterior region of the substantia nigra, but further work is needed to study correlation between DaT-SPECT values, free water values and dopaminergic cell of the nigrostriatal system.

This study has some limitations: (i) Not all enrolled subjects underwent DaT-SPECT; only those in the prodromal stage or with overt DLB did. However, all overt PD patients were expected to have nigrostriatal dopaminergic impairment, and PD diagnosis was confirmed after at least 12 months of clinical follow-up. (ii) DaT-SPECT and MRI were not acquired simultaneously; DaT-SPECT was performed within six months before or after MRI. However, this period is still below the time resolution of DaT-SPECT, since it usually needs 12 to 18 months to detect reliable nigro-striatal pathway's differences. Nonetheless, prodromal patients were clinically evaluated every six months and remained prodromal at the time of the second imaging technique. (iii) The enrolled subjects did not undergo alpha-synuclein seed essay, hence it is possible that within the control group there may be patients with co-occurrence of alpha-synucleinopathy and amyloid disease. Indeed, some of the patients with AD had an absent swallow tail sign. We expect that the statistical significance of the results presented in this study would increase, if patient were grouped based on pathology-proven classification; these considerations warrant future larger multicentric studies with pathological confirmation.

In conclusion, visual analysis of the SMWI swallow tail sign has the potential to become a reliable biomarker for detecting SN neurodegeneration within the continuum of alpha-synucleinopathies, since the prodromal stages of the disease. This approach is applicable at single-subject level, aligning with the new era of biological definitions of alpha-synucleinopathy, and may facilitate earlier diagnosis.

## Supplemental Material

sj-docx-1-pkn-10.1177_1877718X251387027 - Supplemental material for MRI susceptibility map weighted imaging (SMWI) as a neurodegeneration biomarker in the prodromal to overt alpha-synucleinopathy continuum Supplemental material, sj-docx-1-pkn-10.1177_1877718X251387027 for MRI susceptibility map weighted imaging (SMWI) as a neurodegeneration biomarker in the prodromal to overt alpha-synucleinopathy continuum by Laura Falcitano, Francesco Calizzano, Pietro Mattioli, Oliver C Kiersnowski, Laura Avanzino, Nicola Giovanni Girtler, Andrea Diociasi, Mattia Losa, Federico Massa, Silvia Morbelli, Beatrice Orso, Elisa Pelosin, Gaia Bonassi, Stefano Raffa, Matteo Pardini, Mauro Costagli, Luca Roccatagliata and Dario Arnaldi in Journal of Parkinson's Disease
