# Supplementary material for: MRI susceptibility map weighted imaging (SMWI) as a neurodegeneration biomarker in the prodromal to overt alpha-synucleinopathy continuum
Source: J Parkinsons Dis. 2025 Dec 3:1877718X251387027. Online ahead of print. doi: 10.1177/1877718X251387027 (PMC13347558; doi:10.1177/1877718X251387027)
Supplement: Supplementary file 1 [file sj-docx-1-pkn-10.1177_1877718X251387027.docx]

**Title: MRI susceptibility map weighted imaging (SMWI) as a neurodegeneration biomarker in the prodromal to overt alpha-synucleinopathy continuum**

Short title: SMWI swallow tail sign in the alpha-synucleinopathy continuum

Laura Falcitano MD^1#^, Francesco Calizzano, MD^2#^, Pietro Mattioli, MD^1,2*^, Oliver C. Kiersnowski, PhD^1^, Laura Avanzino, MD PhD^1,3^, Nicola Giovanni Girtler PsyD^2,^ Andrea^,^ Diociasi, MD^6^, Mattia Losa, MD^2^, Federico Massa, MD PhD^1,2^, Silvia Morbelli, MD PhD^4^, Beatrice Orso, PhD^2^, Elisa Pelosin, DPT PhD^1,2^, Gaia Bonassi MSC PhD^2^, Stefano Raffa, MD^1^, Matteo Pardini, MD PhD^1,2^, Mauro Costagli, PhD^1,2^, Luca Roccatagliata, MD PhD^1,5,‡^, Dario Arnaldi, MD PhD^1,2,‡^

1IRCCS Ospedale Policlinico San Martino, Genova, Italy

2Department of Neuroscience, Rehabilitation, Ophthalmology, Genetics, Maternal and Child Health, University of Genova, Genova, Italy

3Department of Experimental Medicine, University of Genova, Genova, Italy

4Division of Nuclear Medicine, Department of Medical Sciences, University of Turin, Turin, Italy

5Department of Health Sciences, University of Genova, Genova, Italy

6Department of Internal Medicine and Medical Specialities (D.I.M.I.), University of Genoa

#these authors contributed equally

^‡^ these authors contributed equally

*Correspondence to:

Pietro Mattioli, MD

IRCCS Ospedale Policlinico San Martino, Genova, Italy

Department of Neuroscience, Rehabilitation, Ophthalmology, Genetics, Maternal and Child Health, University of Genova, Genova, Italy

[S3636680@studenti.unige.it](mailto:S3636680@studenti.unige.it)

**Supplementary figure 1.** Schematic representation of the MRI data processing pipeline. Input data consist of magnitude and phase images acquired with a multi-echo GRE sequence, shown on the left side of the figure. Multi-echo magnitude images, yielding T2* contrast, enable the generation of one resultant T2*-weighted image (obtained by averaging across echoes) and the quantitative R2* maps obtained via the ARLO method (1)QSM is obtained from the phase images(2), which carry the information of subtle magnetic field perturbations caused by tissue magnetic susceptibility. Finally, SMWI is obtained by merging the T2*-weighted GRE magnitude and QSM (3).

**Supplementary figure 2.** Segmentation of the SN in one typical subject. The automated segmentation pipeline consists of the application of the “Multi-atlas tool for automated segmentation of brain gray matter nuclei and quantification of their magnetic susceptibility” (4), followed by erosion via a convolution with a 1 mm Gaussian kernel to avoid possible partial volume effects with neighboring tissues. The typical output is shown in sagittal, coronal and axial views (from left to right) superimposed to T2*weighted images (images on the top) and QSM (bottom).

# **Supplementary references**

1.	Pei M, Nguyen TD, Thimmappa ND, Salustri C, Dong F, Cooper MA, et al. Algorithm for fast monoexponential fitting based on Auto-Regression on Linear Operations (ARLO) of data. Magn Reson Med. 2015 Feb 1;73:843–50.

2.	Lancione M, Bosco P, Costagli M, Nigri A, Aquino D, Carne I, et al. Multi-centre and multi-vendor reproducibility of a standardized protocol for quantitative susceptibility Mapping of the human brain at 3T. Physica Medica. 2022 Nov 1;103:37–45.

3.	Nam Y, Gho SM, Kim DH, Kim EY, Lee J. Imaging of nigrosome 1 in substantia nigra at 3T using multiecho susceptibility map-weighted imaging (SMWI). Journal of Magnetic Resonance Imaging. 2017 Aug;46:528–36.

4.	Li X, Chen L, Kutten K, Ceritoglu C, Li Y, Kang N, et al. Multi-atlas tool for automated segmentation of brain gray matter nuclei and quantification of their magnetic susceptibility. Neuroimage. 2019 May 1;191:337–49.
